# Genetic characterization of chicken infectious anaemia viruses isolated in Korea and their pathogenicity in chicks

**DOI:** 10.3389/fcimb.2024.1333596

**Published:** 2024-02-13

**Authors:** HyeSoon Song, HyeonSu Kim, YongKuk Kwon, HyeRyoung Kim

**Affiliations:** Avian Disease Division, Animal and Plant Quarantine Agency, Gimcheon, Republic of Korea

**Keywords:** chicken infectious anaemia virus, complete genome sequencing, pathogenicity, immunosuppression, quantitative real-time PCR

## Abstract

Chicken infectious anaemia virus (CIAV) causes severe anemia and immunosuppression through horizontal or vertical transmission in young chickens. Especially, vertical transmission of virus through the egg can lead to significantly economic losses due to the increased mortality in the broiler industry. Here, 28 CIAV complete sequences circulating in Korea were first characterized using the newly designed primers. Phylogenetic analysis based on the complete sequences revealed that CIAV isolates were divided into four groups, IIa (2/28, 7.1%), IIb (9/28, 32.1%), IIIa (8/28, 28.6%) and IIIb (9/28, 32.1%), and exhibited a close relationship to each other. The major groups were IIb, IIIa and IIIb, and no strains were clustered with a vaccine strain available in Korea. Also, for viral titration, we newly developed a quantitative PCR assay that is highly sensitive, reliable and simple. To investigate the pathogenicity of three major genotypes, 18R001(IIb), 08AQ017A(IIIa), and 17AD008(IIIb) isolates were challenged into one-day-old specific-pathogen-free (SPF) chicks. Each CIAV strain caused anaemia, severe growth retardation and immunosuppression in chickens regardless of CIAV genotypes. Notably, a 17AD008 strain showed stable cellular adaptability and higher virus titer *in vitro* as well as higher pathogenicity *in vivo*. Taken together, our study provides valuable information to understand molecular characterization, genetic diversity and pathogenicity of CIAV to improve management and control of CIA in poultry farm.

## 1 Introduction

Chicken infectious anaemia virus (CIAV), the member of the genus *Gyrovirus* of the family *Anelloviridae*, is a small, icosahedral, non-enveloped virus with a negative-sense, single-stranded, circular DNA genome (Iwata et al., 1998). The viral genome consists of 2.3 kb with three partially overlapping open reading frames (ORFs) for viral protein VP1 (51.6kDa), VP2 (24 kDa), and VP3 (13.6 kDa) (Ducatez et al., 2005; Song et al., 2018). VP1 is the major structural capsid protein associated with viral replication, cell infection ability and virulence. In addition, VP1 is usually used to determine the viral genotype due to its genetic variability (Zhang et al., 2013; Li et al., 2017). VP2 is a scaffolding protein inducing apoptosis and cytopathic effects (CPE) characteristic (Kaffashi et al., 2015). VP1 and VP2 are known to induce the production of neutralizing antibody (NA) in the host (Renshaw et al., 1996; Chowdhury et al., 2003). VP3 is a virulence factor known as apoptin leading to apoptosis in thymocytes and lymphoblastoid cell (Noteborn et al., 1994). The amino acid composition of CIAV is very conservative, with only VP1 presenting significant variability in amino acid positions 139 – 151. Later, it was reported that amino acid at 394 in VP1 could be a major genetic determinant of virulence (Yamaguchi et al., 2001).

CIAV causes severe anaemia, hemorrhaging, and immunosuppression due to depletion of precursor T cells in the thymus and hemoblastocysts in the bone marrow. Immunosuppression enhances the susceptibility to secondary infections and decreases responsiveness to vaccines in young chicks. Yuasa et al. (1979) and Goryo et al. (1985) demonstrated that one-day-old chicks inoculated with CIAV showed severe anaemia with a hematocrit value below 20%, yellowish bone marrow, and marked atrophy of the thymus and bursa of Fabricius between 14 and 16 dpi. McNulty et al. (1990) reported that CIAV developed dose-dependent anaemia in experimentally inoculated chicks even though most of the chicks had sufficient CIAV-specific antibodies. These clinical symptoms occur when chicks are infected during the first two weeks of life but can be avoided if hens transfer sufficient antibodies to their progeny (Tongkamsai et al., 2019a).

CIAV transmission can occur horizontally by direct contact or contaminated fomites, and vertically when seronegative hens become infected during egg production (McNulty et al., 1990; Li et al., 2017).

If the hens are seropositive, maternal antibody generally protects chicks from disease, but not from infection. If mortality occurs, it generally was between 10 and 20%. However, in young broilers, it occasionally reached 60% around three weeks of age. Besides, the loss of net income of approximately 18.5% has been reported due to growth retardation, secondary complications and subclinical disease (McIlroy et al., 1992). Hence, it is necessary to prevent the transmission of CIAV and development of clinical illness in young chicks.

CIAV was first isolated from specific-pathogen-free (SPF) chicks in Japan (Yuasa et al., 1979; Imai and Yuasa, 1990), and then has been commonly identified in the poultry industry worldwide. In Korea, CIAV was found in 1989, and later the molecular characterization of CIAV was analyzed with *VP1* gene in our previous study (Kim et al., 2010). To date, there are many reports on the molecular characteristics of each genotype (Chowdhury et al., 2003; Ducatez et al., 2005; Eltahir et al., 2011; Zhang et al., 2013; Li et al., 2017; Erfan et al., 2018; Yao et al., 2019), but no studies comparing differences in pathogenicity have been reported. In the current study, we elucidated the molecular characterization of CIAV strains circulating in Korea between 2008 and 2019. The genetic diversity of CIAV isolates was analyzed based on the complete sequences. Further, for the first time, pathogenesis study of major genotypes was investigated in chickens to provide guidance for controlling the CIAV transmission.

## 2 Materials and methods

### 2.1 Sample preparation

A total of 28 flocks were collected from different breeds including broilers (n = 9), layers (n = 3), broiler breeders (n = 3), and Korean native chickens (n = 12) between 2008 and 2019 for the presence of CIAV. The flocks ranged in age from 17 to 62 days representing different regions in Korea. Tissue samples (thymus, bone marrow and liver) were homogenized in phosphate-buffered saline (PBS) and viral DNA was extracted using QIAamp DNeasy Blood & Tissue kit (Qiagen, Germany) in accordance with the manufacturer’s directions. CIAV genomic DNA was screened by a BlackCheck CIAV/MDV/REV Multi Detection Kit (Ventech Science, South Korea). For PCR amplification, initial denaturation at 94°C for 5 minutes was followed by 35 cycles of 94°C for 50 seconds, 57°C for 50 seconds, and 72°C for 50 seconds, with a final extension at 72°C for 5 minutes. The CIAV-positive homogenized samples were frozen and thawed three times, and then centrifuged at 3,000 rpm for 20 min. These supernatants were heated at 70°C for 5 min and centrifuged at 3000 rpm for 5 min. The supernatants were filtered through a 0.22-μm filter and added to Marek’s disease virus-transformed lymphoblastoid cell line MDCC-MSB1 in 6-well plates (Van Dong et al., 2020).

### 2.2 Amplification of CIAV genome

For encompassing the complete genome, specific four pairs of primers were designed based on the highly conserved regions of the published CIAV sequences in GenBank: AF285882, AF311900, AY843527, DQ991394, JX260426, KX447637, KY486154, MH001555, MK358456, MK770259, and MN299317.

Specific primers were shown in **Table 1**. PCR assays were performed in a total of 20 μl volume containing 2 μl of each genomic DNA extracted from the cell culture supernatant, 1 μl of each primer (10 pmoles/μl), and BlackPCR Singleplex PCR Premix (Ventech Science, South Korea) with the reaction conditions follows: initial denaturation at 94°C for 5 min, followed by 35 cycles of denaturation at 94°C for 50 sec, annealing at 60°C for 50 sec, and extension at 72°C for 50 sec, and a final elongation at 72°C for 5 min. All PCR assays for each sample were carried out in duplicate. Amplified four PCR fragments were visualized under UV light after separation by 1.5% agarose gel electrophoresis, and sequencing was analyzed by Cosmogenetech Co. (South Korea).

**Table 1:** Oligonucleotide primers for CIAV complete genome sequencing and quantification.

Primer set			Sequence (5’- 3’)	Product size(bp)
Full genome sequencing	I	F	ACTATTCCATCACCATTCTAGCC	1,215
		R	TCCGATTTTGCTCACGTATGT	
	II	F	AACTGCGGACAATTCAGAAAG	1,137
		R	GTGAGT GTTGCAAAGCTCATA	
	III	F	AACGTTCAGTTTCTAGACGGT	1,092
		R	ATTGTAATTCCAGCGATACCAATC	
	IV	F	TCAATGAACCTGACATACGTGA	1,182
		R	TCCATC TTGACTTTCTGTGTACA	
Quantification	F		TTTCAAGAATGTGCCGGACTT	151
	R		GTCTTATACACCTTCTTGCGGT	
	probe		GAAACCCCTCACTGCAGAGAGATC	

### 2.3 Sequence alignment and phylogenetic analysis

The obtained sequences were quality trimmed and aligned using the CLC Main Workbench version 7.6.4 (Qiagen, Germany), with the following steps: removal of low-quality sequence and ambiguous nucleotides. Subsequently, the trimmed sequences were assembled with the CLC Main Workbench software, and the complete genome sequences were compared with the available GenBank sequences to investigate the relationship between genetic divergence.

Phylogenetic analysis was carried out by a neighbor joining (NJ) method using kimura-2-parameter with 1000 bootstrap replication. Relevant VP1, VP2 and VP3 sequences of representative field strains and common vaccine strains collected from GenBank were used for comparison.

### 2.4 Calculation of a standard curve

For evaluation of CIAV titer, the entire *VP2* gene of CIAV was amplified using specific primers (forward primer, 5’- TTATGCACGGGAACGGCGGACA -3’; reverse primer, 5’- TAATCACACTATACGTACCGGGGCGGGG -3’). Two µl of template DNA was added to 18 µl of reaction mixture containing 0.1 mM of each primer and 2 × BlackPCR Singleplex PCR Premix (Ventech Science, South Korea). PCR cycles were as follows: pre-denaturation at 94°C for 5 min, denaturation at 94°C for 40 sec, annealing at 61°C for 40 sec, extension at 72°C for 40 sec, for 35 cycles, extension at 72°C for 5 min. The amplified PCR product was inserted into the pGEM-T Easy vector (Promega, USA), and the recombinant plasmid pGEM-VP2 was confirmed by sequencing. Serial 10-fold dilutions containing copies of 1.0 × 10^8^ ~ 1.0 × 10^1^ were used as templates to generate a standard curve.

Real-time PCR assays were carried out in a 20 μl final volume including 1.0 μl of pGEM-VP2 plasmid, 10.0 μl TaqMan™ Fast Advanced Master Mix (Applied Biosystems Incorporated, CA), 5 μM of each primer and probe in **Table 1**. The qPCR reaction was performed using a QuantStudio 3 Real-Time PCR System (Applied Biosystems, CA) under the following conditions: 95°C for 2 min, then 40 cycles of denaturation for 1 sec at 95°C, annealing and elongation for 20 sec at 60°C. The dilutions were tested in triplicate and used as quantification standards to generate the standard curve by plotting the plasmid copy number logarithm against the measured cycle threshold (Ct) values.

To evaluate the reproducibility of the qPCR assay, three different dilution levels (10^6^, 10^5^, and 10^3^ copies/μl) of the standard plasmid were prepared. Each dilution was performed in triplicate within a single run for measures of intra- and inter-assay variation. Additionally, 28 CIAV genomes were subjected to the qPCR assay.

### 2.5 Animal experiment design

A total of 60 one-day-old specific-pathogen-free (SPF) chicks were equally divided into four groups, G1, G2, G3, and G4. To determine the pathogenicity of three distinct genotypes, 18R001 (genotype IIb, 10^2.5^ TCID_50_/㎖), 08AQ017A (genotype IIIa, 10^3.0^ TCID_50_/㎖) and 17AD008 (genotype IIIb, 10^7.0^ TCID_50_/㎖) strains were intramuscularly inoculated with the highest titer into G1, G2, and G3, respectively. G4 was inoculated with PBS as a negative control.

Three chickens from each group were randomly chosen and individual body weights (BWs) were recorded at 7, 14, 21, and 28 days post infection (dpi). The three chickens were euthanized for collecting thymus, bone marrow, spleen, and bursa of Fabricius. To evaluate the effects of viral infection on growth retardation and immunosuppression, the weight of each thymus was measured. Thymus indexes were calculated according to the following formula: index (mg/g) = (weight of thymus)/body weight × 100%.

The size of thymus and color of the femur bone marrow was assessed, and the representative tissue samples were fixed in 10% neutral buffered formalin for 24 h. Formalin-fixed tissues were processed routinely through graded ethanol, xylene, and paraffin embedding to obtain 5 ㎛ thick sections, and stained with hematoxylin and eosin (H&E) stain for histopathologic evaluation under a light microscope.

Heparinized blood samples were collected from the jugular vein of all chickens at 7-day intervals after inoculation. After centrifugation at 1,200g for 5min, packed cell volumes (PCVs) were determined using the microhematocrit capillary tube method. Chickens can be considered anaemic if their PCV values were < 27.0% according to the previous research (Schat, 2009).

To investigate the viral distribution, each genomic DNA, from thymus, bone marrow, spleen, and bursa of fabricius, was extracted separately using QIAamp DNeasy Blood & Tissue kit (Qiagen, Germany). The extracted DNA was analyzed with qPCR, and values were represented as mean CIAV copy number log_10_ DNA copies/g.

Serum samples were used to monitor the development of CIAV-specific antibodies using a commercially available CIAV ELISA Kit (BioChek, USA). All chicks were confirmed to be free of CIAV antibody at the time of inoculation.

## 3 Results

### 3.1 Virus detection and isolation

All 28 samples were positive for CIAV and listed in the **Table 2**. Of note, no other avian viruses (Avian leukosis virus, ALV; Marek’s disease virus, MDV; avian influenza virus, AIV; infectious bursal disease virus, IBDV; infectious bronchitis virus, IBV; reticuloendotheliosis virus, REV) were detected in these samples (data not shown). After several serial passages in MDCC-MSB1 cells, PCR analysis, with a commercial BlackCheck CIAV/MDV/REV Multi Detection Kit, showed that CIAV were successfully isolated. Of these strains, the highest titer of 18R001, 08AQ017A, and 17AD008 from MDCC-MSB1 cells were about 10^2.5^ TCID_50_/㎖, 10^3.0^ TCID_50_/㎖, and 10^7.0^ TCID_50_/㎖ at 120hr after the start of inoculation.

**Table 2:** Analysis of major variations of amino acid residues derived from VP1 gene of 28 CIAV isolates circulating in Korea.

Strain		Host	Age (week)	Year	Accession NO. in this study	Length(bp)	Genotype	Amino acid positions								
								75	89	125	139	141	144	157	287	394
Majority virulent strain		–	–	–	–	–	–	V	T	I	Q	Q	Q	V	T	Q
Low virulent strain		–	–	–	–	–	–	I	A	L	K	E	E	M	S	H
Reference	Cux-1	–	–	–	–	–	IIIb	V	T	I	K	Q	D	V	A	Q
	Cuxhaven-1	–	–	–	–	–	IIIb	T	I	I	Q	Q	D	V	A	Q
	P4	–	–	–	–	–	IIIb	T	I	I	Q	Q	E	M	T	Q
	Del-Ros	–	–	–	–	–	IIIb	T	I	I	Q	Q	V	M	S	Q
19AQ023		Broiler	4.5	2019	MW091355	2,298	IIa	I	T	I	Q	Q	Q	V	T	Q
09AD367		Broiler	–	2009	MW091356	2,298	IIa	I	T	I	Q	Q	Q	V	T	Q
19AD069		Korea native chicken	6.5	2019	MW091358	2,298	IIb	I	T	I	Q	Q	Q	V	T	Q
19AD028		Layer	8.6	2019	MW091351	2,298	IIb	I	T	I	Q	Q	Q	V	T	Q
19AQ026		Broiler	5.5	2019	MW091353	2,298	IIb	I	T	I	Q	Q	Q	V	A	Q
18R083		Korea native chicken	12	2018	MW091350	2,298	IIb	I	T	I	Q	Q	Q	V	T	Q
18R080		Korea native chicken	10.7	2018	MW091349	2,298	IIb	I	T	I	Q	Q	Q	V	T	Q
18R072		Korea native chicken	8	2018	MW091348	2,298	IIb	I	T	I	Q	Q	Q	V	T	Q
18R014B		Korea native chicken	4	2018	MW091352	2,298	IIb	I	T	I	Q	Q	Q	V	T	Q
18R001		Korea native chicken	6	2018	MW091354	2,298	IIb	I	T	I	Q	Q	Q	V	T	Q
17AD007		Korea native chicken	6	2017	MW091357	2,298	IIb	I	T	I	Q	Q	Q	V	T	Q
19AQ001		Korea native chicken	8.5	2019	MW091342	2,298	IIIa	I	T	I	Q	Q	Q	V	A	Q
18AD027		Broiler	3.1	2018	MW091339	2,298	IIIa	I	T	I	Q	Q	Q	V	A	Q
18R058B		Korea native chicken	9	2018	MW091340	2,299	IIIa	I	T	I	Q	Q	Q	V	A	Q
16AD092		Broiler breeder	14	2016	MW091341	2,298	IIIa	I	T	I	Q	Q	Q	V	A	Q
12AQ041		Broiler breeder	9.2	2012	MW091345	2,298	IIIa	I	T	I	Q	Q	Q	V	A	Q
10AQ095		Broiler breeder	3.1	2010	MW091344	2,298	IIIa	I	T	I	Q	Q	Q	V	A	Q
09AQ183		–	2.6	2009	MW091343	2,298	IIIa	I	T	I	Q	Q	Q	V	A	Q
08AQ017A		Broiler	5.7	2008	MW091346	2,298	IIIa	I	T	I	Q	Q	Q	V	A	Q
19AD011		Layer	4.3	2019	MW239170	2,298	IIIb	V	T	L	K	Q	E	M	S	Q
18R059		Korea native chicken	10	2018	MW091334	2,298	IIIb	V	T	L	K	Q	E	M	S	Q
18AD062		Broiler	2.3	2018	MW091335	2,298	IIIb	V	T	L	K	Q	E	M	S	Q
18AD038		Layer	9	2018	MW091337	2,298	IIIb	V	T	L	K	Q	E	V	S	Q
17AD008		Korea native chicken	9	2017	MW091338	2,298	IIIb	V	T	L	K	Q	E	M	S	Q
10AQ135		Broiler	5	2010	MW091347	2,298	IIIb	V	T	I	K	Q	E	V	S	Q
09AD354		Korea native chicken	7.6	2009	MW091336	2,298	IIIb	V	T	L	K	Q	E	V	S	Q
09AD301		Broiler	4.1	2009	MW091332	2,298	IIIb	V	T	L	K	Q	E	V	S	Q
09AQ174		Broiler	2.3	2009	MW091333	2,298	IIIb	V	T	L	K	Q	E	V	S	Q

(A, Alanine; D, Aspartic acid; E, Glutamic acid; H, Histidine; I, Isoleucine; K, Lysine; L, Leucine; M, Methionine; Q, Glutamine; S, Serine; T, Threonine; V, Valine).

### 3.2 Molecular characterization of 28 CIAV complete genomes

CIAV genome was amplified with four CIAV-specific primer pairs and visualized in four bands on 1.5% agarose gels: a 1,215 bp band for *VP1*, a 1,137 bp band for *VP1*, *VP2* and *VP3*, a 1,092 bp band for *VP1*, *VP2* and *VP3*, and a 1,182 bp band for *VP1*, respectively. After assembling the sequences, the complete *VP1*, *VP2* and *VP3* gene sequences were 1350, 651 and 366 nucleotides long, and the complete sequences were submitted to GenBank (**Table 2**). Of the 28 CIAV isolates, 27 CIAV isolates had 2,298 bp size, while 18R058B was 2,299bp containing G insertion on 210 nucleotide position. This insertion had no influence on the amino acid level of VP1, VP2, and VP3. Previous reports have shown that several amino acid substitutions at positions 75, 89, 125, 139, 141, 144, 157, 287 and 394 of the VP1 protein are related with viral pathogenicity and replication (Renshaw et al., 1996; Yamaguchi et al., 2001; Todd et al., 2002; Ducatez et al., 2005; Yao et al., 2019; Tan et al., 2020). All isolates carried T89, Q141 and Q394, representing the high pathogenicity in the host. At amino acid positions 75 and 125, residue 75 with an isoleucine (I) and residue 125 with a leucine(L) are associated with lower pathogenicity in chickens. Except for 10AQ135, 19 genomes had I75 and I125, and eight genomes had V75 and L125. Four isolates (17AD008, 18AD062, 18R059 and 19AD011) possessed methionine (M) at amino acid position 157 as low virulent strains, while the other isolates carried valine (V) at amino acid position 157 as high virulent strains. In addition, the patterns of amino acid substitutions, located at positions 139, 144, and 287, are reported in **Table 2**. The VP2 and VP3 nucleotide and amino acid sequences were highly conserved in comparison with other strain sequences retrieved from GenBank.

### 3.3 Phylogenetic analysis

**Figure 1:**
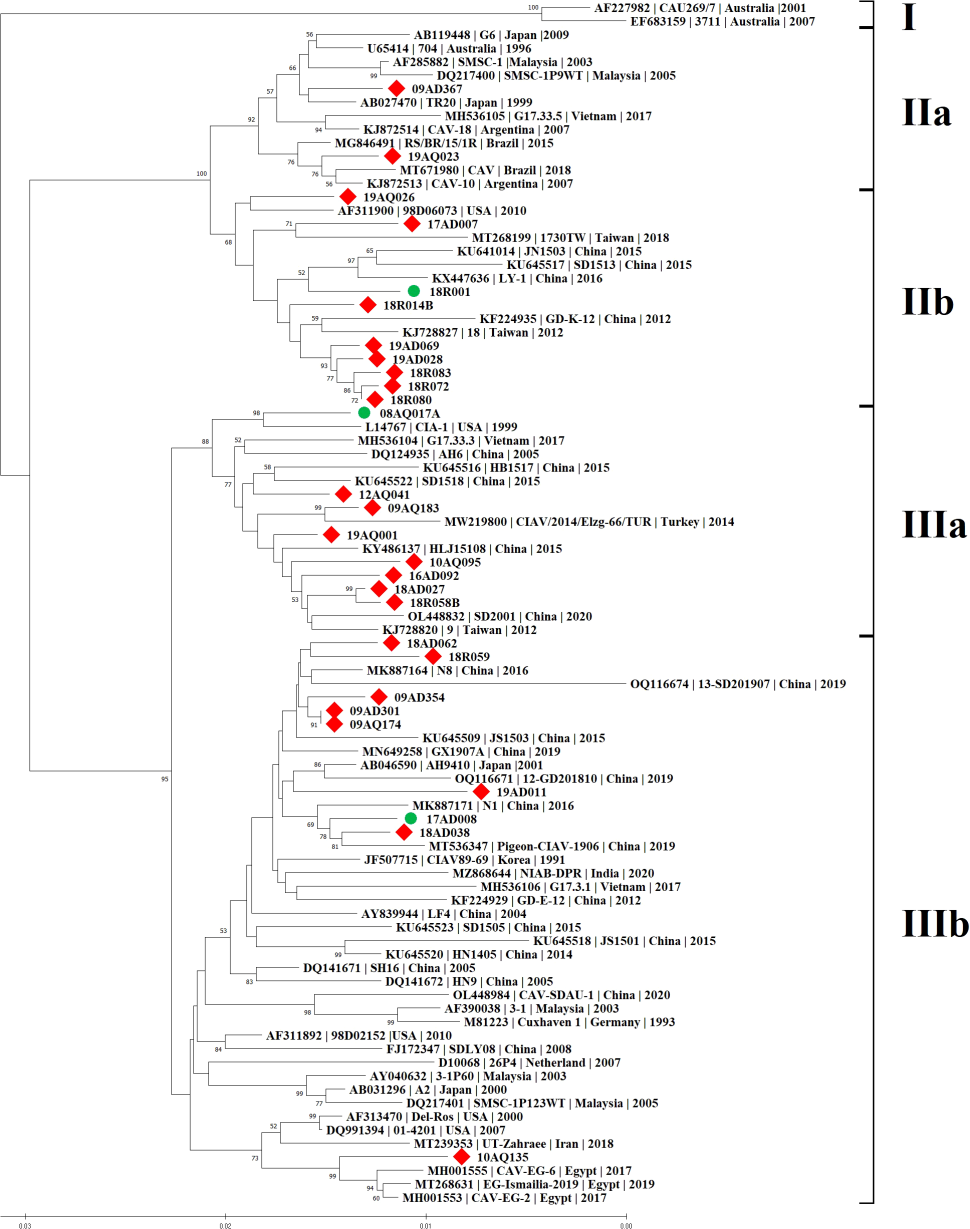
Phylogenetic tree of 28 CIAV complete genome sequences in Korea. The tree was constructed by the Neighbor-Joining (NJ) algorithm and a consensus of 1000 bootstrap replicates. The 25 CIAV isolates are represented by red squares in contrast to the different CIAV isolates from the worldwide listed in GenBank. The three isolates used to investigate the pathogenicity were marked by green circles.

Phylogenetic analysis was carried out based on the complete genome sequences of 28 CIAV isolates and reference strains. CIAV isolates were divided into four groups: IIa (2/28, 7.1%), IIb (9/28, 32.1%), IIIa (8/28, 28.6%) and IIIb (9/28, 32.1%). The major groups were IIb, IIIa and IIIb, and no 28 CIAV strains belonged to group I, which was isolated only from Australia (**Figure 1**). Interestingly, 10AQ135 strain clustered with a vaccine strain Del-Ros, whereas no strains clustered with a vaccine strain 26P4 available in Korea.

Korean CIAV strains showed between their complete sequences a percentage of identity ranging from 95.8% to 100.0% at the nucleotide level. The maximum diversity was 4.2% between 17AD007 of genotype IIb and 19AD011 of genotype IIIb. Both 09AQ174 and 09AD301, which belonged to genotype IIIb, shared 100.0% identity at the nucleotide and amino acid levels. Comparative analyses showed that the 28 genomic sequences presented a 94.9% ~ 99.6% homology with 61 reference strains from GenBank. The maximum diversity was 5.1% between 19AD011 of genotype IIIb, and 3711 (accession no. EF683159, isolated from Australia in 2007) of genotype I. The minimum diversity was 0.4% among 09AQ174, 09AD301 and N8 (accession no. MK887164, isolated from China in 2016) of genotype IIIb (**Supplementary Table S1**).

### 3.4 Establishment of a qPCR

The qPCR amplification curves were generated by using 10-fold serial dilutions of plasmid pGEM-VP2 template. The qPCR assay covered a linear range eight orders of magnitude from 1.0 × 10^8^ copies/μl to 1.0 × 10^1^ copies/μl. Ct values were plotted against the known copy numbers of the standard controls. A correlation coefficient (R^2^) value of 0.998, a slope value of −3.548, an average efficiency value of 91.4% and the Y intercept value of 16.743 [Y = −3.548X+16.743, Y = threshold cycle, X = natural log of concentration (copies/μl)] were obtained (**Supplementary Figure S1A**). Specificity of the optimal qPCR primers was examined using genomic DNA extracted from the different viruses. The primers yielded amplification products only in the reaction tube that contained the specific target genomic DNA (**Supplementary Figure S1B**). The detection limit was 1.0 × 10^2^ copies/μl (**Supplementary Figure S1C**), and this indicates that the qPCR assay was 2.5 times more sensitive than the conventional PCR (Song et al., 2018). The qPCR assay produced a high repeatability with coefficient of variation (CV) within runs (from 0.52% to 0.67%) and between runs (from 0.19% to 0.61%) (**Supplementary Table S2**). A total of 28 CIAV strains were probed using the qPCR assay. The amplification results were 100% in agreement with those of the PCR using four pairs of CIAV-specific primers and a commercial BlackCheck CIAV/MDV/REV Multi Detection Kit (**Supplementary Table S3**). Therefore, these results showed that the qPCR assay is highly sensitive, specific and reproducible.

### 3.5 Gross lesions and histopathological changes

**Figure 2:**
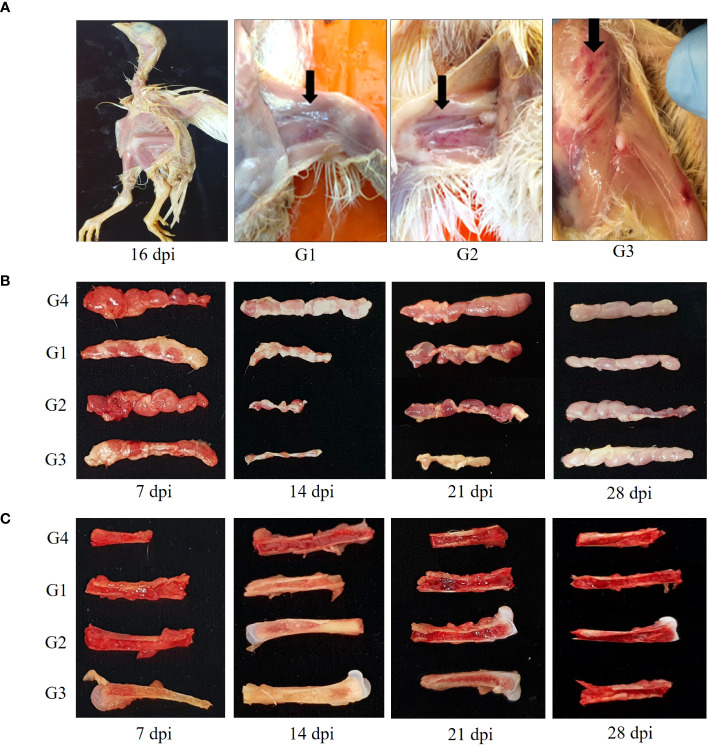
Gross lesions change in chickens. **(A)** Severe growth retardation in G3 at 16 dpi and hemorrhages presented in thigh, leg and breast muscles in CIAV-infected groups at 14 dpi. **(B)** Atrophied thymic lobules infected with 18R001 (G1), 08AQ017A (G2), and 17AD008 (G3) compared to a control group (G4) at 14 dpi. **(C)** Yellowish or red bone marrow in CIAV-infected and non-infected groups at 14 dpi.

Several chicks in G1, G2, and G3 showed depression, reluctance to move, growth retardation and generalized weakness from 14 dpi to 18dpi. Mortality reached 13.3% (2 of 15) in G3 at 16 dpi. At 14 dpi, the postmortem examination revealed petechial hemorrhage of the thigh, leg and breast muscles, severe thymus atrophy, and weaken femurs with pale aplastic bone marrow (**Figures 2A–C**). As shown in **Figure 2B**, thymus in G1, G2 and G3 began to appear smaller after 7 dpi, and showed a 50 to 80% reduction in size relative to control thymus at 14dpi. Thymus lesions in G3 appeared as marked reduction in size up to 21 dpi.

Red bone marrow in G1, G2, and G3 transitioned to yellow bone marrow after 7 dpi and the production of new blood cells becomes more difficult at 14 dpi (**Figure 2C**). Atrophied thymus and pale bone marrow in G1, G2 and G3 gradually returned to normal at 28 dpi.

**Figure 3:**
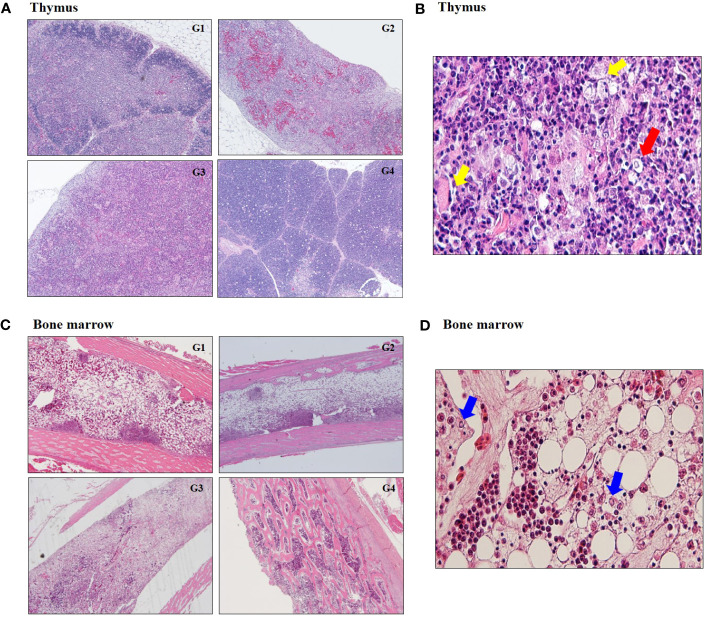
Histopathological changes at 14 dpi. **(A)** Representative image of severe atrophy and absence of demarcation between cortex and medulla in thymus infected with 18R001 (G1), 08AQ017A (G2), and 17AD008 (G3) compared to a control group (G4). **(B)** Image of hydropic swelling (yellow arrow) of epithelial cells and enlarged nuclei (red arrow) in thymus infected with CIAV. **(C)** A prominent decrease in the number of hemopoietic cells in the bone marrow. **(D)** Image of multiple small eosinophilic inclusion bodies (blue arrow) in macrophage.

In histopathology examination, thymus in G1, G2 and G3 showed the reduction in the size of the thymic cortex due to severe cortical thymocytes depletion and absence of demarcation between the medulla and cortex (**Figure 3A**; **Supplementary Table S4**). There was hydropic swelling of epithelial cells and enlarged nuclei at 14 dpi (**Figure 3B**). Bone marrow in G1, G2 and G3 exhibited severe depletion of hematopoietic lineages (**Figure 3C**). There was highly significant decrease in heterophil and increase in lymphoblast depletion in G1, G2, and G3 regardless of CIAV genotype (**Figure 3D**; **Supplementary Table S4**).

### 3.6 T/B ratio change

**Figure 4:**
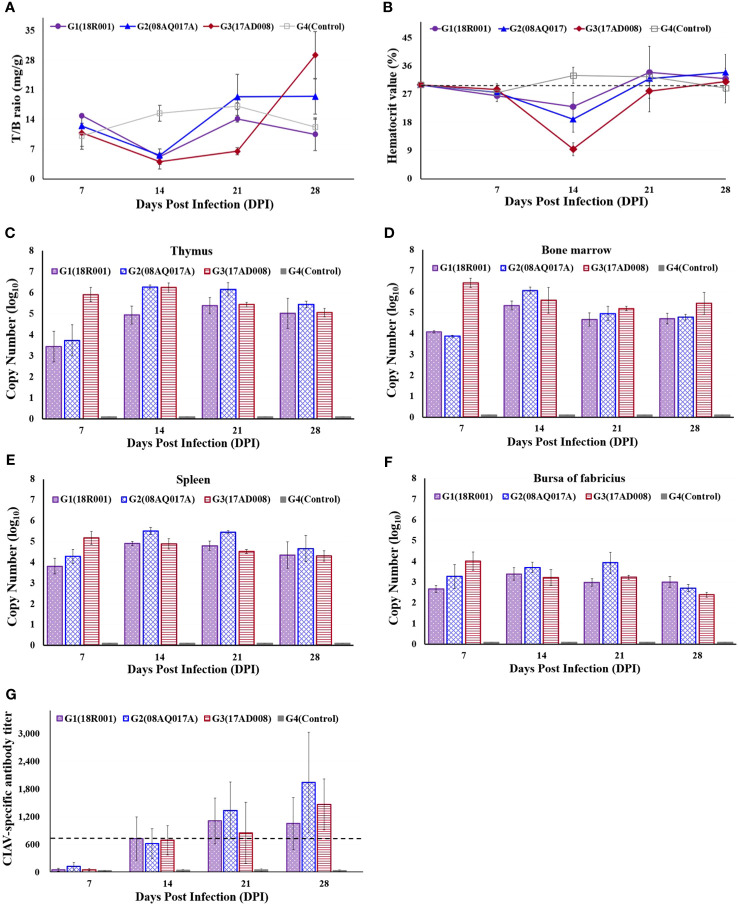
Comparison of the ratio of thymus to body weight, packed cell volumes (PCV), viral distribution in immune organs and CIAV-specific antibody levels in CIAV-infected and non-infected groups. **(A)** Thymus index in each group. **(B)** Detection of PCVs at different time points. A PCV value below 27% was regarded as anemia. **(C–F)** Viral distribution in thymus, bone marrow, spleen, and bursa of Fabricius, respectively. **(G)** Dynamic detection of CIAV-specific antibody levels. A titer value below 724 was considered CAV antibody negative.

The thymus to body weight ratio was measured. The relative thymus weights in G1, G2, and G3 were lower than those in the control at 14 dpi. At 21 dpi, the mean thymus to body weight ratios in G3 were still lower than those of the control chickens. However, in G1, G2, and G4, the difference with respect to each other was not significant (**Figure 4A**).

### 3.7 Hematological change

The average PCVs were given below; ranged from 23.0% to 34.0% in G1, between 19.0% and 34.0% in G2, and ranged from 9.5% to 31.0% in G3. PCVs in G1, G2, and G3 were significantly lower than that of the control group at 14dpi, and in G3, PCV value was 9.5%, indicating the severe anaemia.

After 14 dpi, PCVs have increased, showing more than 27.0% (**Figure 4B**). No anaemic birds were detected in the G4.

### 3.8 Virus distribution in tissues

Viral loads were estimated in thymus, bone marrow, spleen, and bursa of Fabricius at 7, 14, 21, and 28 dpi. CIAV was all positive from 7 dpi to 28 dpi. Although a general downward trend of the viral copy number in G1, G2 and G3 was from 14 dpi, the viral loads were still high on 28 dpi (**Figures 4C–F**). No CIAV was detected in G4.

### 3.9 Serologic change

CIAV-specific antibody levels were determined at 7, 14, 21, and 28 dpi. At 7dpi, all samples were negative, and then have significantly increased in the CIAV-infected groups. In G1, the antibody levels reached a maximum level at 21 dpi, while all chickens in G2 and G3 had the highest antibody levels at 28 dpi (**Figure 4G**). The control group maintained a negative status during the experiment.

## 4 Discussion

For analyzing viral genotype with complete sequences, four pairs of CIAV-specific primers were newly established, and genetic diversity of 28 CIAV isolates was analyzed. Although several CIAV-specific primers were designed in the previous studies (Zhang et al., 2013; Li et al., 2017), no primers actually amplified a specific region (from nucleotide position 2,165 to 2,251) with GC contents of 70.0% or more in 28 CIAV isolates. Here, four pairs of CIAV-specific primers successfully amplified the individual target regions, and the region (from nucleotide position 2,165 to 2,251) was completely generated with 1182 and 1092 primers. The obtained complete genome sequences had a high level of nucleotide similarity with each other, and the divergence is only 0.0% (09AD301 and 09AQ174) to 4.2% (17AD007 and 19AD011). Only one CIAV isolate (18R058B) had one nucleotide insertion in a non-coding region. Analysis of amino acid sequences in VP1 protein showed that the substitution at residue 287 was a threonine (T), serine (S) or alanine (A). Among the 28 CIAV isolates, 10 strains carried a T as the majority of virulent strains, and nine strains had a S as low virulent strains. However, the remaining nine isolates possessed an A, whose function has not been identified. Likewise, in China, only one out of 15 CIAV strains had an A that has not been identified in other strains (Yao et al., 2019).

Whole genome phylogenetic analysis resolved a total of four distinct groups (IIa, IIb, IIIa, and IIIb) with significantly higher bootstrap values. Most of the CIAV isolates belong to genotypes IIb, IIIa and IIIb. Interestingly, 10AQ135 was clustered with a commercial vaccine strain, and the nucleotide identity between 10AQ135 and Del-Ros displayed 98.6%. In Korea, a commercial vaccine Del-Ros has not been available, and no strains like 10AQ135 have been discovered since 2010. Thus, it is a probability that 10AQ135 might be transmitted through SPF eggs imported from abroad.

To determine the relationship between DNA copy numbers and infectivity titration, CIAV titers were estimated in MDCC-MSB1 cells, as previously described (Imai and Yuasa, 1990; Yamaguchi et al., 2000; Tseng et al., 2019). The newly developed qPCR assay was validated with the recombinant plasmid pGEM-VP2 containing the entire *VP2*. *VP2*, a phosphatase, is considered to assist VP1 protein folding during viral particle assembly (Liu et al., 2023). The qPCR assay indicated that the average Ct value of 12.67 corresponds to the 1.0 x 10^8^ plasmid DNA copies or 10^7.0^ TCID_50_/㎖ (data not shown). Viral titration by the traditional infectivity titration method required more than 24 days, but the qPCR assay took only 35 min. Therefore, the qPCR assay proved to be more robust, quicker and easier to interpret than conventional TCID_50_ assessment approaches.

To examine the association between virulence and genotype characteristics, three CIAV isolates, 18R001 (IIb), 08AQ017A (IIIa), and 17AD008 (IIIb), were selected to investigate pathogenicity within three major genotypes. These strains exhibited visible cytopathic effects (CPE) in MDCC-MSB1 cells (**Supplementary Figure S2**). Each CIAV isolate yielded varying sensitivities and replication rates on MDCC-MSB1 cells, with the highest titers of 10^2.5^ TCID_50_/㎖, 10^3.0^ TCID_50_/㎖, and 10^7.0^ TCID_50_/㎖. Viral replication rates vary among infected cells, possibly as a result of variation in viral genome sequences or via differences in the host cell (Bruurs et al., 2023). In the previous report, some CIAV showed differential sensitivity depending on the MSB1 cell subtype, and glutamine (Q) at the amino acid positions 139 and 144 could decrease the rate of virus replication and the spread of CIAV (Renshaw et al., 1996). 18R001 and 08AQ017A carried Q at the amino acid positions 139 and 144, while 17AD008 possessed lysine (K) at position 139 and glutamic acid (E) at position 144. Consequently, certain amino acid variations can play an important role in cellular tropism, within-host spread and pathogenicity between different CIAV isolates.

After inoculation of these three isolates into one-day-old chicks, most of the chicks infected with CIAV developed anaemia, and bone marrow appeared fatty or yellowish at 14 dpi. The results are in agreement with those reported by Yuasa, Goryo and McNulty (Yuasa et al., 1979; Goryo et al., 1985; McNulty et al., 1990). Especially, in G3, PCV values at 14 dpi were <10%, which indicated the severe anaemia, and the mortality was 13.3%. Besides, the apparent decrease of body weight and thymus index was observed from 14 to 21. Atrophy of the thymus was more conspicuous in the G3 than in G1 and G2. About half of yellow bone marrow was gradually replaced by red bone marrow in G1 and G2 at 14 dpi, while the color change slowly appeared in G3 at 21 dpi. These differences between G1, G2, and G3 seem to be more affected by the viral titer than viral genotype. Surviving chicks completely recovered from anaemia and depression after 21 dpi.

In G1, G2, and G3, all 180 tissue samples (thymus, bone marrow, spleen, and bursa of Fabricius) were positive for CIAV on qPCR analysis. The presence of high viral load levels was observed at the same time as the production of antibodies in the CIAV-infected groups. Although CIAV-specific antibody titer was increased in the serum, viral replication was still observed in all tissues until the end of the experiment. Previous studies have reported that lymphoid tissues are the primary targets for CIAV infection, and CIAV persists in thymic lymphocytes where it cannot be neutralized by the antibody (Cardona et al., 2000; Markowski-Grimsrud and Schat, 2003; Tongkamsai et al., 2019a). Continuous dissemination of the virus from the thymus with insufficient antibody response and the release of infected cells from the thymus into blood circulation might be the cause for the increased virus distribution in the other tissues (Tongkamsai et al., 2019b). Subsequently, CIAV can be replicated in the secondary tissues, and transmitted through horizontal contact or vertical transmission via hatching eggs. Hence, our results suggest that it is impossible to eradicate CIAV under field conditions once the virus is introduced into a chicken flock. The chicken flock with CIAV-induced immunosuppression can be exposed to other infections, leading to reduce production performance. CIAV coinfection with other pathogens, such as Marek’s disease virus (MDV), infectious bursal disease virus (IBDV), reticuloendotheliosis virus (REV), avian leukosis virus (ALV), and fowl adenovirus (FAdV), has been documented to enhance the disease severity and epidemiology in previous studies (Zhang et al., 2021; Zhang et al., 2022). So, it is important to monitor the presence of CIAV antibody in breeder flocks to avoid vertical transmission. In Korea, to prevent the vertical transmission from breeders to progeny, breeder flocks are frequently vaccinated with an imported live-virus vaccine. Vaccination is an effective way to control CIAV and prevent outbreaks of the disease.

In our further study, to develop a new vaccine for Korean strains, a subsequent passage process in MDCC-MSB1 cells is essential to attenuate 17AD008. Several previous studies regarding attenuation have been reported. Bülow and Fuchs (1986) stated the attenuation of Cux-1 was found after 49 passages (P49) in cell culture, while Todd et al. (1998). showed that Cux-1 became considerably less pathogenic at P170 in MDCC-MSB1 cells. Chowdhury et al. (2003). indicated that two Malaysian CIAV strains were partly attenuated at p60 and at P123, respectively.

In conclusion, CIAV strains circulating in Korea were successfully isolated in cell culture, and each complete genome sequence was generated using newly designed primers. Also, for viral titration, a simple, reliable and useful qPCR assay was newly established in this study. Three major genotypes CIAV strains caused anaemia and immunosuppression in chickens. In particular, the novel 17AD008 strain is distinguished from other CIAV strains for its stable cellular adaptability and high viral titers in MDCC-MSB1 cells. This work provides the method and strategies in experiments for virus isolation, molecular characterization, genetic diversity, quantitative titration and pathogenicity of CIAV. These findings serve as a foundation for future directions to control the horizontal and vertical spread of CIAV in poultry farms.

## Patents

Two patent numbers, 1025563160000 (12 July 2023) for a method for determining the complete sequences of CIAV, and 1020210053458 (26 April 2021) for a method for determining viral titers, were approved by the Korean Intellectual Property Office (KIPO).

## Data availability statement

The original contributions presented in the study are included in the article/**Supplementary Materials**, further inquiries can be directed to the corresponding author/s.

## Ethics statement

The animal study was approved by Animal Ethics Committee of the Animal and Plant Quarantine Agency. The study was conducted in accordance with the local legislation and institutional requirements.

## Author contributions

HS: Conceptualization, Data curation, Formal analysis, Writing – original draft. HSK: Data curation, Investigation, Methodology, Writing – original draft. YK: Writing – review & editing. HRK: Writing – review & editing.
