# Supplementary figures and images for: Genetic characterization of chicken infectious anaemia viruses isolated in Korea and their pathogenicity in chicks
Source: Front Cell Infect Microbiol. 2024 Feb 13;14:1333596. doi: 10.3389/fcimb.2024.1333596 (PMC10900523; doi:10.3389/fcimb.2024.1333596)

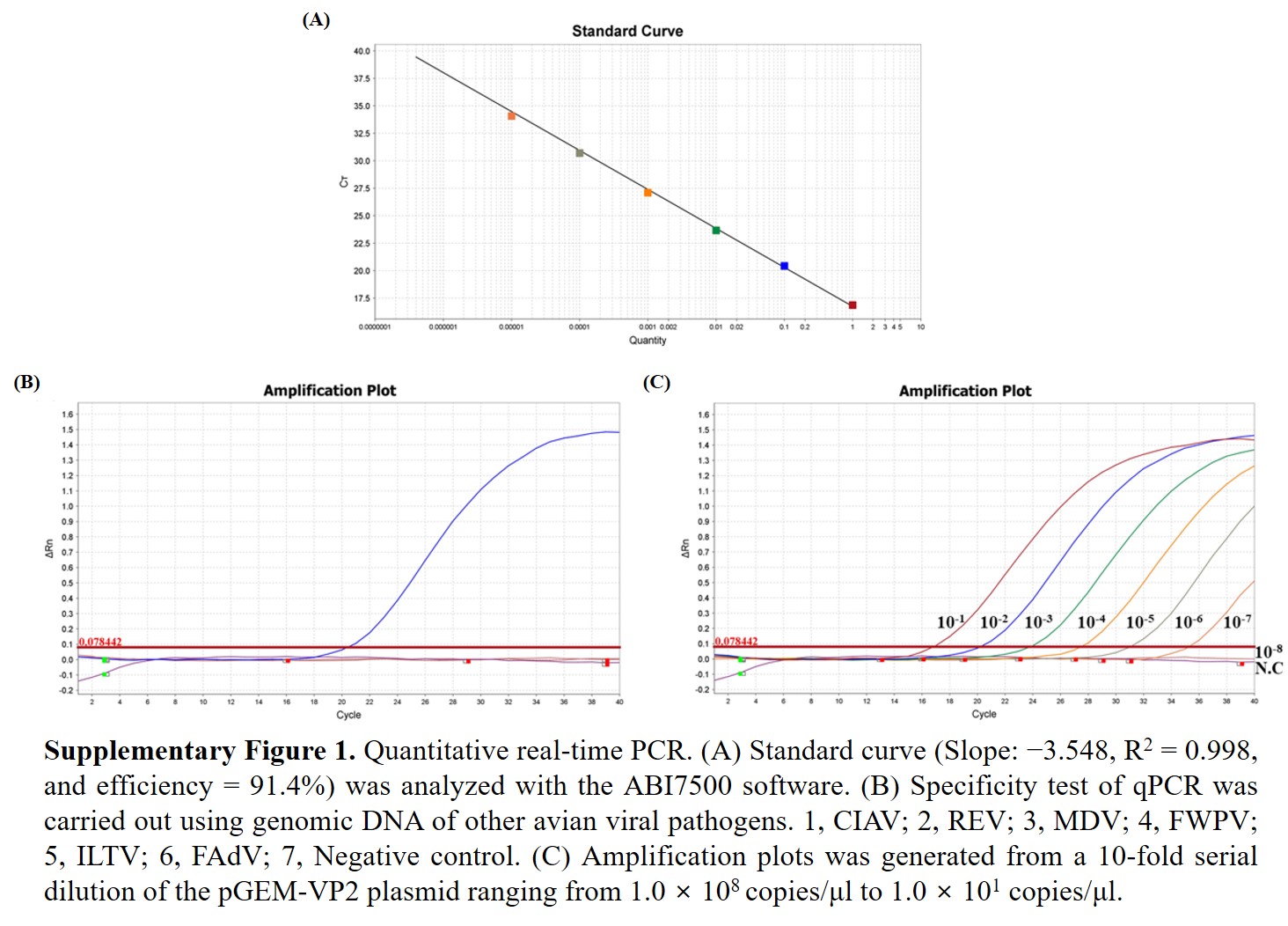

Supplement: Supplementary file 1 [file Image_1.jpeg]

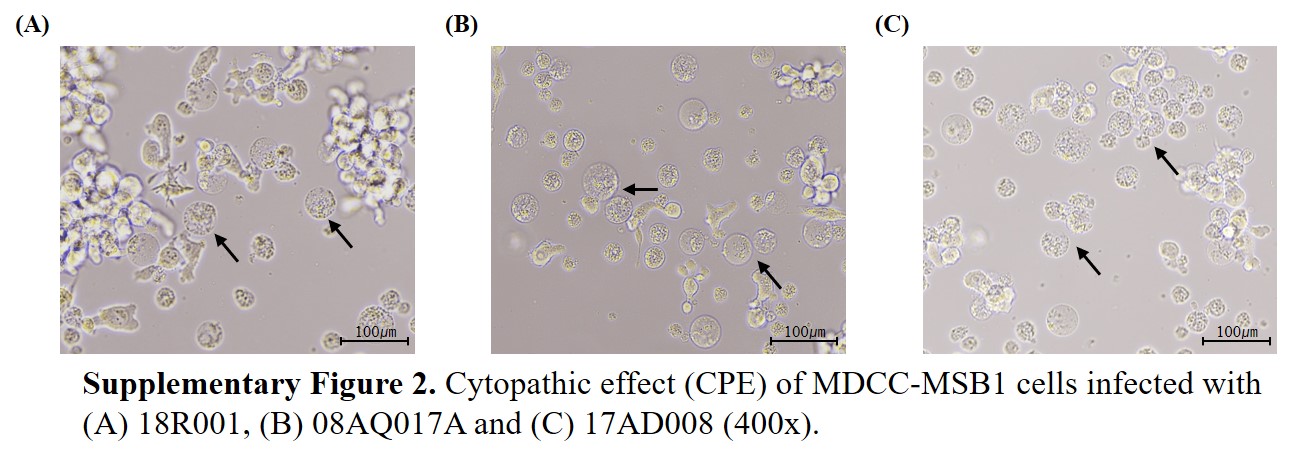

Supplement: Supplementary file 2 [file Image_2.jpeg]
